# Predicting surgical outcomes for chronic exertional compartment syndrome using a machine learning framework with embedded trust by interrogation strategies

**DOI:** 10.1038/s41598-021-03825-4

**Published:** 2021-12-20

**Authors:** Andrew Houston, Georgina Cosma, Phillipa Turner, Alexander Bennett

**Affiliations:** 1 School of Computer Science, Loughborough University, Loughborough, LE11 3TU UK; 2 Academic Department of Military Rehabilitation, Defence Medical Services, Loughborough, LE12 5QW UK; 3 Centre for Lower-Limbs Rehabilitation, Defence Medical Services, Loughborough, LE12 5QW UK; 4 Imperial College London, National Heart and Lung Institute, London, SW7 2BU UK; 5 School of Sport, Exercise and Health Sciences, Loughborough University, Loughborough, LE11 3TU UK

**Keywords:** Biomarkers, Prognostic markers, Medical research

## Abstract

Chronic exertional compartment syndrome (CECS) is a condition occurring most frequently in the lower limbs and often requires corrective surgery to alleviate symptoms. Amongst military personnel, the success rates of this surgery can be as low as 20%, presenting a challenge in determining whether surgery is worthwhile. In this study, the data of 132 fasciotomies for CECS was analysed and using combinatorial feature selection methods, coupled with input from clinicians, identified a set of key clinical features contributing to the occupational outcomes of surgery. Features were utilised to develop a machine learning model for predicting return-to-work outcomes 12-months post-surgery. An AUC of 0.85 ± 0.08 was achieved using a linear-SVM, trained using 6 features (height, mean arterial pressure, pre-surgical score on the exercise-induced leg pain questionnaire, time from initial presentation to surgery, and whether a patient had received a prior surgery for CECS). To facilitate trust and transparency, interrogation strategies were used to identify reasons why certain patients were misclassified, using instance hardness measures. Model interrogation revealed that patient difficulty was associated with an overlap in the clinical characteristics of surgical outcomes, which was best handled by XGBoost and SVM-based models. The methodology was compiled into a machine learning framework, termed AITIA, which can be applied to other clinical problems. AITIA extends the typical machine learning pipeline, integrating the proposed interrogation strategy, allowing to user to reason and decide whether to trust the developed model based on the sensibility of its decision-making.

## Introduction

Chronic exertional compartment syndrome (CECS) is a condition occurring most frequently in the lower limbs^1–4^ and is prevalent in individuals who partake in activities such as walking, running and marching whilst carrying load^5^. Symptoms related to CECS include severe pain in the affected compartment, occurring around 15 min after the onset of exercise^6,7^. For military personnel, particularly infantry soldiers, this presents a challenge and can often result in an inability to perform their job.

Surgical interventions in the form of compartment-specific fasciotomies have become a prominent method of treating CECS^8,9^. Whilst fasciotomies have proven successful amongst civilian populations, enabling more than 75% of athletes to return to sport^10–15^, the same cannot be said for military personnel who have been described as having less reliable outcomes^16^. Audits suggest that less than 45% of UK military personnel manage to return to a fully-fit state^17^, with most recent evidence showing this number to be as low as 22%^18^.

Despite the problem of poor outcomes in military populations, only one study, to date, has sought to identify reasons as to why patients fail to have good surgical outcomes^19^. Waterman et al.^19^ applied binary logistic regression analysis to a large dataset of active military personnel, to identify variables that are associated with surgical failure. Results revealed significant associations between surgical failure and perioperative complications, activity limitations and the chronicity of the CECS. Although factors that are associated with surgical outcomes were identified, the predictive value of these factors was not evaluated. Furthermore, whilst methods such as logistic regression are suitable for identifying relationships and performing basic predictions, through the application of machine learning, generalised linear models can be outperformed^20^.

The application of machine learning within orthopaedic medicine is a growing area, showing promise in treatment outcome prediction^20,21^. However, despite the promising status of machine learning, literature applying it to tackle orthopaedic problems remains relatively surface-level limiting the evaluation of models to global performance estimates such as overall accuracy and area under the receiver operating characteristic curve (AUC)^22–24^. The use of global estimates of performance makes it impossible to understand how a model performs on patients across the difficulty spectrum. Patient difficulty is derived from instance hardness, a term first defined by Smith et al.^25^ as the likelihood of an instance being misclassified and can be the result of factors such as class overlap, i.e. where the characteristics of each treatment outcome or group overlap^25,26^. Clinically, this definition can be translated as a patient whose presentation and outcome do not align, posing a unique challenge in machine learning problems. Given that classifiers employ different decision functions, their suitability for handling difficult patients will vary depending on the source of difficulty^27^. Furthermore, to instigate trust in developed models, an analysis of patient difficulty and its source can help to examine whether misclassifications arise from sensible assumptions of the model or whether they are a result of bias present within the dataset. From an applicability perspective, trust is imperative in the design of machine learning models for healthcare prognosis, and so, understanding the decision-making process of a prediction model is imperative to instil said trust.

This paper presents the development of a machine learning model to predict the return-to-work outcomes of military personnel following a corrective fasciotomy for CECS, using routinely collected pre-surgical patient data from a military rehabilitation facility. Combinatorial feature selection methods were coupled with the domain knowledge of clinicians to identify the best predictors. Thereafter, a battery of machine learning models were evaluated for the classification task. The computational novelty of this paper is the interrogation of model performance that was used to facilitate trust in the model development process characterising difficult patients using instance hardness, identifying the source of difficulty that was most affecting classification performance and identifying which models were best equipped to deal with this challenge. Lastly the proposed methods are compiled into an Artificial Intelligence with Trust by InterrogAtion framework termed, AITIA, for developing and interrogating the machine learning models to identify the trustworthiness of their decision-making.

## Results

Throughout this paper, data associated with a single surgery will be referred to as a ‘record’. The database of the Defence Medical Information Capability Program was queried against the inclusion-exclusion criteria defined in the ‘Online methods’ section, returning 132 records from 119 patients. Of the 132 records, a total of 6 records were identified as being outliers and were removed, resulting in a final dataset containing 97 records with a poor surgical outcome and 29 records with a good surgical outcome. Post-processing the dataset contained 23 features and the descriptive statistics, grouped according to outcome, are presented in Supplementary Table S2.

### Statistical analysis

To determine whether the dataset has been drawn from a normally distributed population the normality of each feature was assessed using the Kolmogorov–Smirnov and Shapiro–Wilk tests. Results revealed a non-normal distribution across the majority of features in this dataset (Supplementary Table S1. Therefore, non-parametric tests were used throughout.

To identify the between-group differences between surgical outcomes, a Mann–Whitney U test was performed on the ordinal data and Chi-square test on the categorical data (Supplementary Table S2). The results of the Mann–Whitney U test showed that patients with a successful outcome were significantly younger (Z = − 1.972, p = 0.049), taller (Z = − 2.717, p = 0.007) and had a lower body mass index (BMI) (Z = − 2.179, p = 0.029) than patients with an unsuccessful outcome. Patients with a successful outcome also had significantly shorter chronicity (Z = − 4.071, p < 0.001), time from presentation to treatment (TTT) (Z = − 4.657, p < 0.001), time from presentation to diagnosis (TTD) (Z = − 4.579, p < 0.001), and time spent medically downgraded (TDG) (Z = − 3.628, p < 0.001). Results of the Chi-square tests showed an association between successful outcomes and both lack of inpatient rehabilitation prior to surgery ($\chi ^2$ = 6.133, p = 0.018) and previous surgeries for CECS ($\chi ^2$ = 4.865, p = 0.041).

**Figure 1:**
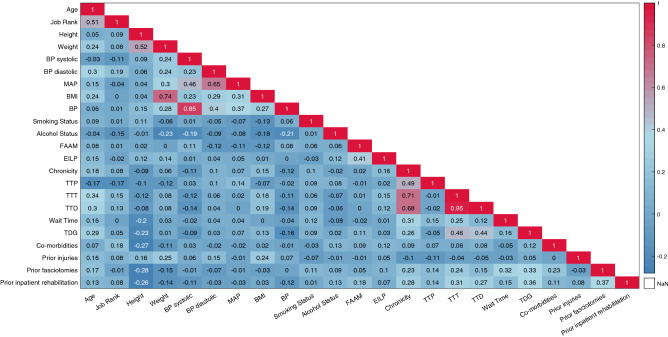
The Spearman’s rank correlation matrix used to identify co-linear features within the dataset. Cells with darker shades of red reflect a positive relationship between the two features associated with that cell, and cells with darker shades of blue reflect a negative relationship between the two associated features. A correlation of 1.0 indicates a perfect positive correlation, and a correlation of − 1.0 indicates a perfect negative correlation. Relationships with a correlation coefficient of greater than 0.5 were deemed to be co-linear and were accounted for when generating the candidate feature sets.

Spearman’s rank correlation was applied to measure the strength of association between pairs of features within the dataset and to identify co-linearities within the dataset (Fig. 1). The inclusion of co-linear features can result in an unpredictable variance of a model’s performance. Results revealed the following 8 co-linear pairs, defined by a correlation coefficient greater than 0.5: age and rank ($\rho$ = 0.507), height and weight ($\rho$ = 0.521), weight and BMI ($\rho$ = 0.854), diastolic blood pressure and mean arterial pressure (MAP) ($\rho$ = 0.654), systolic blood pressure and general blood pressure ($\rho$ = 0.846), chronicity and TTT ($\rho$ = 0.710), chronicity and TTD ($\rho$ = 0.682), and TTD and TTT ($\rho$ = 0.953). Co-linearity will be considered in the selection of statistical and domain knowledge derived feature subsets being tested in the final model, and a minimum redundancy, maximum relevancy fitness function used in the genetic algorithm-based feature selector.

Using the results of the Mann–Whitney U and Chi-square tests, 9 feature sets were created using three alpha thresholds ($\alpha =$ 0.05, 0.01, 0.001), including all features where significance exceeded each threshold, avoiding co-linear pairs being included in the same feature set (Supplementary Table S3).

### Feature selection

To identify a small set of clinically relevant features that are predictive of the surgical outcomes, feature selection was applied. Aside from identifying features relevant to the classification problem, feature selection reduces the dimensionality of the dataset, simplifying the problem, thereby improving model stability and generalisability. Feature selection was performed using a filter-based tabu asexual genetic algorithm (TAGA)^28^ and the knowledge of clinical experts.

As previously mentioned the results of the statistical analysis of the dataset was used to generate 9 features sets. The TAGA then generated an additional 9 features sets, shown in Supplementary Table S3. The optimally performing feature set from each approach was identified by ranking the performance of each feature set, in terms of AUC, for each classifier, and taking the feature set with the lowest summed rank (Supplementary Table S4). The features included in the optimally performing statistically-derived feature set (Stats 6) and the optimal performing TAGA-derived feature set (TAGA 9) were presented to the clinical team who created a final feature set (STAT + TAGA + Expert) comprised of height, MAP, pre-surgical score on the exercise-induced leg pain questionnaire (EILP), TTT, TDG and prior surgeries. The performance of the feature set derived by the clinical teams was determined for each classifier.

The best performing subset for each classifier was identified using AUC (Supplementary Table S4). Where ties existed, the feature set which performed optimally across all classifiers was chosen. For all models, barring the sequential model, STAT + TAGA + Expert proved optimal. The sequential model performed best using Stats 6.

### Classification performance

The classification performance of each tuned model, using their optimal feature set is presented in Table 1. A Friedman’s test was carried out to identify whether performance significantly differed between models for each metric and post-hoc comparisons were carried out using a Wilcoxon signed-rank test. A summary is presented below, with full results shown in Supplementary Table S7.

The support vector machine (SVM) offered the best classification performance in terms of accuracy (0.80 ± 0.07). In comparison with other models, SVM proved significantly better than *k*-nearest neighbours (KNN), random forest, the ensembled KNN and the sequential model, achieving accuracy scores of on average 6%, 3%, 4% and 2% higher than each model, respectively, when evaluated on the test set. With regards to sensitivity, the ensembled KNN proved best at identifying those who returned to work within 12-months of surgery (0.83 ± 0.16). Compared to other models, the ensembled KNN’s classifications were significantly more specific than SVM (+ 2%), random forest (+ 9%), extreme gradient boosting (XGBoost) (+ 11%) and the sequential model (+ 5%). That said, despite the ensemble KNN proving to be highly sensitive, its performance in terms of specificity was the second weakest of the tested models (0.74 ± 0.10), demonstrating it was not as capable of accurately identifying records of patients that had good surgical outcomes. The inverse can be said for XGBoost, which was the most specific model (0.82 ± 0.08), performing significantly better than all other models, however, it was the weakest model in terms of sensitivity (0.72 ± 0.20).

With regards to both AUC and the true positive rate at the optimal point of the receiver operating characteristic curve (TPR), the SVM performed the best, achieving an AUC of 0.85 ± 0.08 and TPR of 0.81 ± 0.12. Compared to the remaining models, SVM’s AUC was significantly higher than all remaining models, barring the ensembled SVM. Furthermore, SVM’s TPR was significantly greater than both KNN (+ 4%) and random forest (+ 3%). Logistic regression had the lowest false positive rate at the optimal point of the receiver operating characteristic curve (FPR) (0.19 ± 0.09), which was significantly lower than KNN (− 5%), random forest (− 5%) and the sequential model (− 2%).

### Machine learning model interrogation

The purpose of this analysis is to interrogate the performance of each of the 8 developed machine learning models, providing reasoning for misclassified records and to understand how each model performs on more difficult records, in addition to performance on the dataset as a whole.

**Figure 2:**
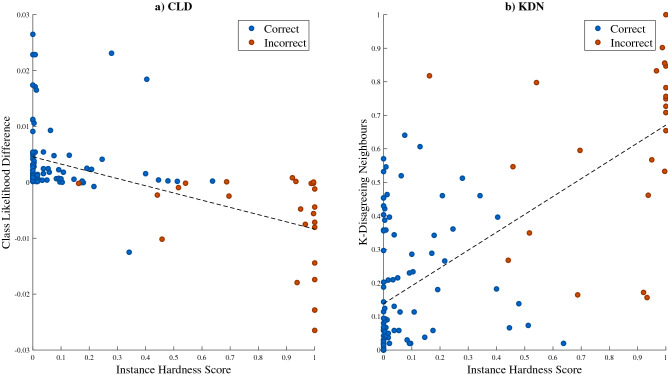
Results of the misclassification analysis applied to the SVM model’s performance demonstrating the relationship between class overlap and instance hardness. As class overlap increases, denoted by a reducing CLD and increasing KDN, instance hardness increases, resulting in more misclassifications by the SVM model.

A Spearman’s rank correlation was applied to identify which hardness measures were strongest associated with instance hardness and therefore, best explain the source of misclassifications in the data. Results revealed a moderate positive correlation between instance hardness and *k*-disagreeing neighbours (KDN) ($\rho$ = 0.661), and a moderate negative correlation between instance hardness and class likelihood difference (CLD) ($\rho$ = − 0.595). Demonstration of these correlations is presented in Fig. 2. Both disjunct size (DS) and disjunct class percentage (DCP) showed moderate negative correlations with instance hardness ($\rho$ = − 0.532 and − 0.524, respectively). Therefore, results demonstrate that the primary reason for misclassifications was due to globalised and localised class overlap, due to KDN and CLD being the two measures, most correlated with instance hardness.

**Figure 3:**
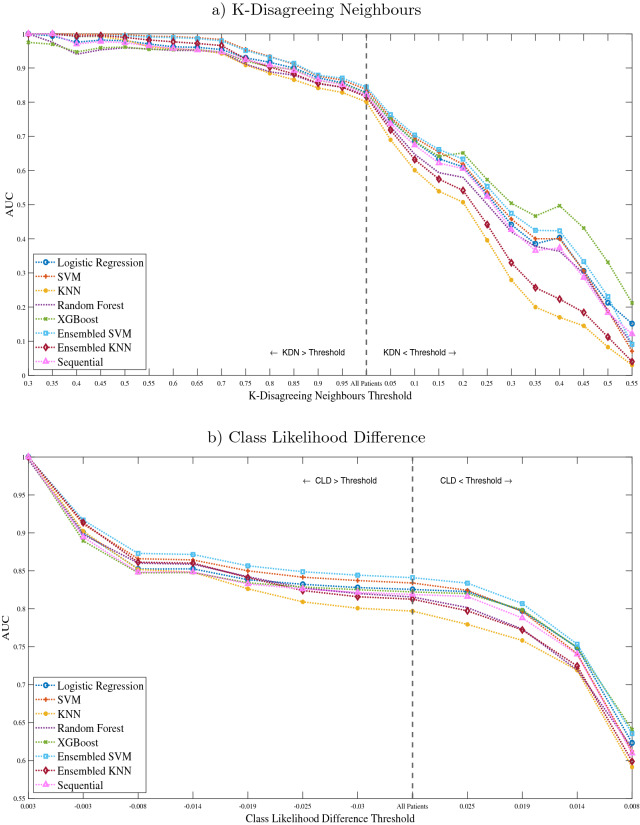
Results of the misclassification analysis showing the effect of increasing class overlap, determined using (**a**) KDN and (**b**) CLD, on the AUC of each model. Each line reflects the AUC of a model, determined using the mean probability of all records surpassing the threshold shown in the X-axis, where points towards the left represent performance on a set of exclusively easy records and those on the right represent performance on a set of exclusively difficult records.

To examine each model’s performance on records of high and low difficulty, AUC was calculated using the mean probability predicted for each record from the 30 iterations of the nested cross-validation, incrementally adding or removing records based on their KDN and CLD scores. This process was conducted, firstly with the least difficult records, characterised as having a KDN score < 0.3 and a CLD score > 0.0027, incrementally adding records with higher KDN scores and lower CLD scores. When all records were included, the least difficult records were incrementally removed until only the most difficult records remained (KDN > 0.5 and CLD < − 0.0191). The thresholds for the least and most difficult records were due to the requirement of having both classes present in the dataset to calculate AUC, and so, when the threshold increased/decreased to where only one class remained, this was deemed to be the limits of the analysis. The results of this process demonstrated that XGBoost and random forest performed marginally worse than the remaining models on records of low difficulty. As difficulty increases, XGBoost demonstrated superior performance than the remaining models (Fig. 3). In contrast, both KNN models show weaker performance on more difficult records (Fig. 3). Both SVM-based models and the logistic regression, perform similarly regardless of record difficulty and therefore in a single-classifier model make for good candidate classifiers.

### Proposed artificial intelligence with trust by interrogation (AITIA) framework

**Figure 4:**
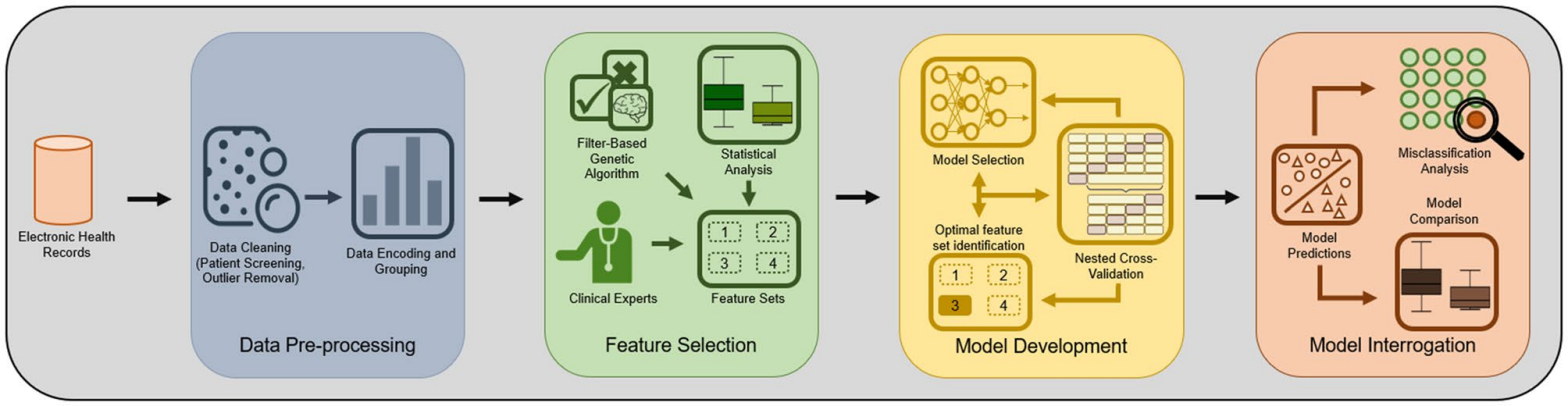
AITIA: An extended form of the traditional machine learning framework with integrated ‘trust by interrogation’ strategies that characterise the difficulty of each record, provide reasoning for individual record misclassification and give an insight into how each model performs on records of varying degrees of difficulty.

A common avenue for facilitating trust in AI solutions is to improve the transparency of a model’s decision making by embedding interpretability measures, such as feature importance, feature contributions and counterfactual explanations. However a recent evaluation of the effects of such measures found that whilst existing methods are successful in improving an end user’s understanding, minimal evidence supported the idea that they empower the end-user to trust a model appropriately^29^. A needs analysis, conducted by Tonekaboni et al. revealed the desire of clinicians to understand on which patients an AI solution performs poorly, recognising that most models are unlikely to be perfect^30^. Quantification of these areas of poor performance and associated sources may go some way to engender trust in a developed model. Based on the outcomes of the experiments performed in this study, identifying the primary source of misclassifications and evaluating the performance of different models across the difficulty spectrum, the methods were compiled into an Artificial Intelligence with Trust by InterrogAtion framework, termed AITIA (Greek: cause and reason). The AITIA framework extends the typical machine learning pipeline of data cleaning $\rightarrow$ pre-processing $\rightarrow$ feature selection $\rightarrow$ model development and evaluation, by including a novel model interrogation process. A diagram of the proposed framework is presented in Fig. 4.

The rationale for the inclusion of clinicians in the feature selection process stems from the idea that their knowledge can provide valuable insights that may be missed by computational methods. Contrarily, the sole reliance on the knowledge of experts can adversely affect model performance through the introduction of bias^31^. The proposed framework combines the knowledge of clinicians with combinatorial feature selection methods, producing a set of features to be tested. This approach capitalises upon the knowledge of clinicians, whilst mitigating the risks of unwanted bias.

The model interrogation process was motivated by the fact that dataset-level measures of model performance cannot reveal why errors occur. Furthermore, the demand for trustworthy machine learning models is unlikely to be satisfied without a robust means of evaluating why a model predicts some patients incorrectly. Although a perfect model would be ideal, this is oftentimes unrealistic, particularly in fields such as orthopaedic medicine where treatment outcomes can be affected by a multitude of factors. Therefore, model interrogation was used to assess whether to trust the decision-making process of the model, even when its prediction is wrong. The code for performing the model interrogation proposed in this study is publicly available at https://github.com/andrewhouston113/Machine-Learning-Model-Interrogation.

### Comparison with automated frameworks

Given the time and costs associated with including domain expertise in the development process of a predictive model, it is important to understand whether this inclusion results in a performance lift compare to automated approaches, thereby, justifying the additional commitment. This section compares the performance of the AITIA framework (Fig. 4) to three well-known and publicly available automated machine learning frameworks, to identify whether a framework with domain knowledge results in better classification performance.

Given the superiority of SVM in terms of accuracy, AUC and TPR (Table 1), in addition to it being both sensitive and specific, with a low FPR and proving capable of handling difficult records (Fig. 3), the SVM was identified as the optimal classifier produced by the proposed framework. Therefore, the performance of the SVM-based model was compared to three automated frameworks—a Tree-based Pipeline Optimization Tool (TPOT)^32^, Auto-Sklearn^33^ and AutoPrognosis^34^—using a Wilcoxon signed-rank test on each performance metric for each of the test sets. The 150 test sets, derived using the methods described in the “Performance comparison with automated machine learning frameworks” section of the ‘Online methods’, were used to evaluate the performance of each automated framework were identical for all frameworks, enabling a fair comparison.

Auto-Sklearn was shown to be the most accurate and specific model, achieving an accuracy score of 0.83 ± 0.07 and a specificity score of 0.89 ± 0.08, although sensitivity was limited (0.62 ± 0.22). Whilst Auto-Sklearn outperformed the proposed method in this paper from an accuracy perspective, the automated approaches have failed to generate a solution capable of accurately identifying records of patients who had a good surgical outcome, denoted by the low sensitivity. Class imbalance was present in the data set, having 29 and 97 records of patients with good and poor surgical outcomes, respectively. Experimentally, it was found that by applying an up-sampling method, in this case, SMOTE, the effects of class imbalance, notably low sensitivity, were mitigated (Supplementary Table S6 vs. Table 1). The assumption could therefore be made that the methods included in each of the automated frameworks were insufficient in dealing with the class imbalance in the dataset, and as such, are unable to identify the records in the minority class with a high degree of accuracy (Table 2). In terms of the other performance metrics, the proposed framework outperformed all other automated approaches across AUC, FPR and TPR (Table 2).

**Table 1:** Results of the nested cross validation for each model, trained using their respective optimal feature set.

Model	Set	Accuracy	Sensitivity	Specificity	AUC	Fpr	Tpr
Logistic regression	Train	0.81 ± 0.03	0.83 ± 0.05	0.81 ± 0.03	0.86 ± 0.03	**0.18 ± 0.03**	0.82 ± 0.04
	Validation	0.79 ± 0.06	0.79 ± 0.15	0.79 ± 0.07	0.84 ± 0.08	**0.21 ± 0.10**	0.80 ± 0.12
	Test	0.80 ± 0.07	0.81 ± 0.18	0.80 ± 0.08	0.84± 0.09	**0.19 ± 0.09**	0.81 ±0.11
SVM	Train	**0.83 ± 0.04**	0.86 ± 0.06	0.82 ± 0.04	**0.88 ± 0.03**	0.16 ± 0.04	**0.84 ± 0.05**
	Validation	**0.80 ± 0.06**	0.80 ± 0.15	0.80 ± 0.08	**0.84 ± 0.08**	0.20 ± 0.11	**0.80 ± 0.11**
	Test	**0.80 ± 0.07**	0.80 ± 0.17	0.80 ± 0.08	**0.85 ± 0.08**	0.20± 0.11	**0.81 ± 0.12**
KNN	Train	0.77 ± 0.04	0.88 ± 0.06	0.74 ± 0.05	0.89 ± 0.03	0.19 ± 0.04	0.82 ± 0.06
	Validation	0.74 ± 0.07	0.82 ± 0.13	0.72 ± 0.09	0.81 ± 0.09	0.24 ± 0.08	0.78 ± 0.12
	Test	0.74 ± 0.08	0.81 ± 0.15	0.72 ± 0.10	0.80± 0.09	0.24 ± 0.08	0.77 ± 0.11
Random Forest	Train	0.79 ± 0.04	0.80 ± 0.08	0.79 ± 0.05	0.86 ± 0.03	0.21 ± 0.04	0.80 ± 0.05
	Validation	0.77 ± 0.07	0.72 ± 0.17	0.78 ± 0.09	0.83 ± 0.08	0.24 ± 0.08	0.76 ± 0.11
	Test	0.77 ± 0.08	0.74 ± 0.20	0.78 ± 0.10	0.82± 0.09	0.24 ± 0.09	0.78 ± 0.12
XGBoost	Train	0.81 ± 0.03	0.73 ± 0.07	**0.83 ± 0.03**	0.85 ± 0.03	0.20 ± 0.04	0.79 ± 0.05
	Validation	0.80 ± 0.06	0.69 ± 0.16	**0.83 ± 0.07**	0.83 ± 0.07	0.22 ± 0.09	0.78 ± 0.11
	Test	0.79 ± 0.07	0.72 ± 0.20	**0.82 ± 0.08**	0.83 ± 0.09	0.20± 0.10	0.79 ± 0.1
Ensembled SVM	Train	0.82 ± 0.04	0.85 ± 0.06	0.81 ± 0.04	0.87 ± 0.03	0.17 ± 0.04	0.83 ± 0.04
	Validation	0.80 ± 0.06	0.80 ± 0.14	0.79 ± 0.08	0.84 ± 0.08	0.20 ± 0.10	0.81 ± 0.12
	Test	0.80 ± 0.07	0.81 ± 0.16	0.79 ± 0.08	0.85± 0.09	0.19 ± 0.11	0.80 ± 0.12
Ensembled KNN	Train	0.78 ± 0.04	**0.89 ± 0.06**	0.75 ± 0.05	0.89 ± 0.03	0.17 ± 0.04	0.83 ± 0.04
	Validation	0.76 ± 0.07	**0.84 ± 0.13**	0.73 ± 0.09	0.83 ± 0.08	0.21 ± 0.09	0.79 ± 0.12
	Test	0.76 ± 0.08	**0.83 ± 0.16**	0.74 ± 0.10	0.82 ± 0.08	0.21 ± 0.10	0.80 ±0.12
Sequential	Train	0.81 ± 0.03	0.83 ± 0.06	0.80 ± 0.04	0.87 ± 0.03	0.19 ± 0.03	0.81 ± 0.04
	Validation	0.78 ± 0.06	0.77 ± 0.16	0.79 ± 0.07	0.83 ± 0.08	0.22 ± 0.09	0.79 ± 0.11
	Test	0.78 ± 0.07	0.78 ± 0.17	0.78 ± 0.09	0.83± 0.09	0.21 ± 0.09	0.78 ± 0.11

Cells highlighted in bold reflect the model identified as being superior with respect to the outcome measure associated with that cell.

**Table 2:** Comparison of the proposed framework compared to automated machine learning frameworks.

Method	Accuracy	Sensitivity	Specificity	AUC	FPR	TPR
AITIA (proposed)	0.80 ± 0.07	**0.80 ± 0.17**	0.80 ± 0.08	**0.85 ± 0.08**	**0.20 ± 0.11**	**0.81 ± 0.12**
TPOT	0.72 ± 0.09	0.49 ± 0.22	0.79 ± 0.12	0.71 ± 0.11	0.24 ± 0.14	0.64 ± 0.13
Auto-Sklearn	**0.83 ± 0.07**	0.62 ± 0.22	**0.89 ± 0.08**	0.82 ± 0.10	0.21 ± 0.15	0.77 ± 0.12
AutoPrognosis	0.78 ± 0.09	0.65 ± 0.22	0.82 ± 0.12	0.80 ± 0.11	0.24 ± 0.14	0.75 ± 0.13

Cells highlighted in bold reflect the framework identified as being superior with respect to the outcome measure associated with that cell.

## Discussion

### Clinical relevance of selected features

The results of the feature selection process and identification of the optimal model/feature set combinations revealed 6 features that proved optimal across all models, barring the sequential model. This is a strength of this study in that the selected features are not biased towards a particular classifier, owing to their promise as markers of surgical outcomes. These features were height, MAP, EILP, TTT, TDG and prior surgeries.

With regards to the use of height as a predictive feature, anatomical findings have shown height to be a discriminatory variable between patients with CECS and healthy controls, with CECS patients being shorter in stature^35,36^. Whilst the literature remains undecided as to whether this is an aetiological factor for the development of CECS, the findings of this study justify that rationale, given that the anatomical predisposition to CECS would likely result in higher recurrence rates, hampering return-to-work prospects.

Evidence has shown lifestyle factors to be associated with musculoskeletal pain^37–39^ and are intrinsically linked with the development of hypertension^38,40^. Given that MAP was identified as valuable for outcome prediction, patients with higher blood pressure could be less likely to return to work due to lifestyle factors inhibiting them from making a full recovery. Additionally, they may be less engaged in post-surgical rehabilitation. However, how MAP relates to surgical outcomes in CECS, specifically, is yet to be determined.

Only one other study to date has identified risk factors for surgical failures in military CECS patients, suggesting that failure rates were higher in patients with high levels of chronicity and physical activity limitations^19^. In concurrence, the present study also found similar outcomes, with TTT and TDG being identified as predictive of surgical outcomes. Reasoning behind TTT outperforming general chronicity as a predictor likely lies in the poor reporting of symptom onset, with patients often estimating when their symptoms started, rather than being able to provide an actual date. The inclusion of prior surgeries as a predictor is supported by the work of Waterman et al.^19^, which showed only 14% of surgical revisions in military personnel go on to experience a full resolution of symptoms.

### Model performance and interrogation

The optimal model, developed using the proposed framework was the SVM, achieving an accuracy score of 0.80 ± 0.07 and AUC of 0.85 ± 0.08. The findings of the model interrogation demonstrate the misclassifications that occur are most often the result of class overlap, given that KDN and CLD were the two measures shown to be most associated with instance hardness ($\rho$ = 0.661 and − 0.595, respectfully). This speaks to the sensibility of the model’s decision making, in that, when errors are made, this is due to those records being more aligned with the opposite outcome, rather than from improperly learning the trends in the data. This finding in combination with the selected feature’s alignment with the literature, allow the user to trust that the model’s decision-making is sensible.

Further to examining the performance of the developed models, the proposed model interrogation can shed light on strategies to improve model performance. Class overlap is oftentimes cited as a primary reason for the increasing difficulty of a record^25^ and as demonstrated in this and other studies is handled poorly by KNN-based models^41^. The model interrogation also revealed that XGBoost was adept in handling difficult records, but was weaker with easier records. Given that different models proved optimal at each end of the difficulty spectrum, methods such as multiple classifier systems^42^ or hybrid trees^43^ may be suitable techniques for improving classification performance. In Termenon et al.^44^ a two-stage sequential ensemble was employed, whereby the first classifier made a prediction, giving an estimation of uncertainty, instances exceeding a given threshold of uncertainty were then passed to a second classifier, specialised in handling more complex instances. Findings showed this approach to achieve superior performance than single classifier systems. Similarly, dynamic classifier selection approaches, which select the most appropriate classifier or ensemble of classifiers for the feature space an instance falls within, have been shown to achieve superior performance in harder instances^41^. Future investigations into the application of multiple classifier systems to clinical problems such as this, with a focus on maintaining acceptable levels of trust and interpretability, should be evaluated.

### Limitations and future works

The primary limitation of this study is the dataset that was used to train and test the developed models. Having only 132 records, restrictions were likely placed on the classification performance capable of being achieved with so few data points. As a result, it is evident that the developed models suffer from a degree of variability that is to be expected when sample sizes are smaller. Furthermore, whilst the results of a deep learning model are presented, it was always unlikely that this approach would prove optimal, given deep learning’s dependency on data^45^. However, it is expected that as the dataset increases in size, these limitations will be minimised.

Secondly, the dataset used in this study is comprised of retrospective data from a single medical facility and therefore limits the generalisability of the findings of this study and the applicability of the developed model. Furthermore, the surgery for which the data refers to were performed by only a 5 different surgeons, all using similar surgical techniques. In that respect, the developed model is suited for the setting in which is was developed. In considerations of these limitations, prior to deployment in a real-world setting, external validation would be required to confirm the selected features and quantify model performance on a truly unseen, prospective set of patients.

Lastly, our comparison with automated frameworks demonstrated the superiority of AITIA. But before this finding can be generalised, additional experiments will need to be conducted, comparing the performance of the frameworks across additional datasets. Furthermore, AITIA provides a novel model interrogation strategy designed to improve an end users level of trust in the model development process. However, an end user evaluation was not included as part of this work and should form the focus of future investigations. Therefore, future works include: (1) validating the performance of the developed model in a prospective group of patients, independent to the data used in this study. (2) an investigation comparing the model interrogation outputs with explainable methods such as feature importance, feature contributions and counterfactual explanations to establish how its inclusion influences an end users trust in and understanding of a model’s decision making. (3) a study to compare the proposed framework to other automated and non-automated frameworks across multiple datasets.

### Implications and conclusions

This study provided an interrogation of a real-world medical dataset, comprised of military CECS patients undergoing surgical intervention. The contributions of this study are both clinical and computational. Clinically, results highlight key patient characteristics that should be considered when referring a patient for a fasciotomy for CECS. The slow referral speed for surgery was identified as a key modifiable feature that was useful in discriminating between good and poor surgical outcomes, and should be a focus for care improvement. Whilst the presented findings must be prefaced with the need for further prospective validation, the presented model could enable better treatment planning and overall improvement of surgical outcomes.

Computationally, this study proposed a machine learning framework, shown in Fig. 4, that: (1) identifies a small set of clinically relevant features that are predictive of the treatment outcome, combining the knowledge of clinicians with computational approaches to achieve an informed result; (2) proposes a model for predicting CECS using the selected features; (3) proposes strategies for interrogating the performance of machine learning models using a misclassification analysis providing potential reasons as to why certain patients cannot be correctly classified and identifies which models are best suited for dealing with challenging patient profiles, facilitating the planning of strategies for improving model performance; (4) proposes AITIA, a framework with embedded ‘trust by interrogation’ strategies that can be used for any clinical problem requiring machine learning.

## Methods

### Dataset description

The raw dataset contained the following 19 features, taken at the time of surgery: age, job rank, height, weight, systolic and diastolic blood pressure ($BP_{systolic}$ and $BP_{diastolic}$, respectively), cigarettes smoked/day, units drank/week, scores of the Foot and Ankle Ability Measure (FAAM) and Exercise Induces Leg Pain questionnaire (EILP), date of symptom onset, date of first presentation to the medical pathway, date of diagnosis, date the patient had first been medically downgraded, whether the patient had any prior injuries, surgeries, inpatient rehabilitation or co-morbidities, a disidentified surgeon ID, and the patient’s treatment outcome.

All methods were carried out in accordance with relevant guidelines and regulations. Data was collected and anonymised by the clinical teams at the Defence Medical Rehabilitation Centre (DMRC) before being provided to the research team. Due to the retrospective nature of the study, informed consent was waived by Ministry of Defence Research Ethics Committee. Approval was granted for the use of the data contained in the database by the clinical director and Caldicott Guardian of the Defence Medical Rehabilitation Centre. CC1 approval was gained for the publication of this study (CC1-20210174).

### Inclusion and exclusion criteria

The Defence Medical Information Capability Program was queried against the following inclusion and exclusion criteria: records of patients were included if they had received a corrective fasciotomy, between 01/01/2014 and 31/12/2019, following a positive diagnosis for CECS, using the criteria specified in Roscoe et al.^46^, defined as an intramuscular compartment pressure of greater than 105 mmHg, during an exercise protocol consisting of carrying a 15-kg backpack, the treadmill incline increased to 5%, and walking pace set at 6.5 km/h for 5 min. Records were excluded if they had refused surgery, encountered a new lower-limb injury the year post-surgery, received a second surgery within the year following the first, or left the military less than 1 year post-surgery.

### Surgical outcome definition

Records were split into two groups according to their treatment outcome, which was used as the classification target. A successful outcome was defined as a return to full deployability status within 1 year post-surgery and an unsuccessful outcome was defined as a sustained status of ‘medically not deployable’ or ‘limited deployability’. Given that a patient may injure another area of their body within that time frame, only the lower-limb portion of the deployability status was considered. One year has been recommended as the optimal follow-up time given that it allows sufficient time to recover from surgery, as well as enabling the identification of re-occurrences^47,48^. Deployability status was assessed by a local medical officer or the occupational medical board and judged against the UK Joint Service Medical Deployment Standards (JSP-950).

### Outlier detection and removal

Outlier identification and removal were employed to ensure that trained models were more capable of appropriately characterising the data. Failure to remove outliers could result in their presence skewing the data distributions, resulting in misrepresentation of the relationships contained within the dataset. To detect outliers, all features were firstly min–max normalised to avoid the impact of features with large ranges biasing the distance calculation. An outlier score was calculated for each record using a relative density approach^49^, reflecting the “outlierness” of a record relative to their nearest 10 neighbours. A threshold of 1.5 interquartile ranges above the upper quartile of outlier scores was the cut-off point for removing records.

### Grouping of features

The raw features were grouped according to public health guidelines, published literature or data distribution (Supplementary Table S2). The motivation for grouping features was due to the nature of the dataset being small and therefore requiring simplification of the contained data to improve the robustness of the trained models and prevent overfitting.

*Age* was binned into three groups based on the distribution of the dataset. The groups were age $\le$ 25, 25 < age $\le$ 30 and age > 30. *Job rank* is already ordinal, however, the frequencies of ranks greater than 3 become sparse and could introduce bias into the predictions, hence jobs ranks of 2 and greater were collectively grouped to account for this. *Height* and *weight* were grouped into three and four groups, respectively, based on the distribution of the dataset. For *height*, groups were height < 170 cm, 170 cm $\le$ height, < 180 cm, height $\ge$ 180 cm. For *weight*, groups were weight < 75 kg, 75 kg $\le$ weight, < 85 kg, 85 kg $\le$ weight, < 95 kg, weight $\ge$ 96 kg. *BMI* was calculated using height and weight, and grouped according to the recommendations of the World Health Organisation^50^ and National Heart, Lung, and Blood Institute^51^.

$BP_{systolic}$ and $BP_{diatolic}$ were grouped into four and three groups respectively, using the thresholds outlined by the American Heart Association^52^. A third variable was created giving an overall categorisation of blood pressure (BP) as ‘Normal’, ‘Elevated’, ‘Stage 1 high BP’ or ‘Stage 2 high BP’, according to the thresholds outlined by the American Heart Association^52^. *Mean Arterial Pressure* (MAP) was calculated using $BP_{systolic}$ and $BP_{diatolic}$ and split into categories of MAP < 60, 60 $\le$ MAP < 100 and MAP $\ge$ 100 which have been identified as predisposing patients to greater risks during and following surgical procedures^53,54^.

In determining the categories of *smoking*, this study looked to literature relating to smoking and musculoskeletal injuries^55,56^, and smoking and general health^57–59^. Heavy smoking was generally regarded as over 20 cigarettes per day^55,58,59^ and a review of the health effects of light and moderate smoking^57^ revealed that generally light smoking in musculoskeletal conditions was classified as < 10 cigarettes per day^55,56^. Therefore, smoking was categorised into ‘Non-Smoker’, ‘Light’ (< 10/day), ‘Moderate’ (10–20/day) and ‘Heavy’ (> 20/day). *Alcohol* is measured in units per week and was binarised into ‘Binge drinker’ (>14 units/week) and ‘Non-binge drinker’ ($\le$14 units/week) to reflect the United Kingdom alcohol consumption guidelines^60^.

Both the FAAM^61^ and EILP^62^ questionnaires are patient-reported outcomes that are routinely used in clinical settings that manage CECS patients, and measure the impact of musculoskeletal pain on a patient’s daily life and were grouped according to the categories incorporated in the questionnaires themselves; extremely affected (< 20%), very affected (20% to < 40%), moderately affected (40% to < 60%), slightly affected (60% to < 80%) and unaffected ($\ge$ 80%). FAAM and EILP were the only two variables to have missing values, with 20% and 22% of records missing pre-surgical values, respectively. Missing data were imputed by applying group-level median imputation, replacing the missing value with the median of the treatment outcome group to which that record belongs. Features relating to co-morbidities, prior injuries, prior fasciotomy and prior inpatient rehabilitation were binarised into ‘Yes’ and ‘No’, due to the low number of records having more than one in any of these variables.

Six pathway variables relating to the timings of the injury and interventions were calculated in months, as follows:

- Chronicity: time from date of symptom onset to date of surgery
- Time-to-Present (TTP): time from date of symptom onset to date of first presentation to a medical facility
- Time-to-Diagnose (TTD): time from date of first presentation to a medical facility to date of diagnosis
- Time-to-Treat (TTT): time from date of first presentation to a medical facility to date of surgery
- Time Downgraded (TDG): time from date of medical downgrade due to symptoms to date of surgery
- Wait Time: time from date of diagnosis to date of surgery

Features relating to the timings of the injury and interventions were grouped following consultation with the clinical lead for CECS at DMRC Stanford Hall and guided by the dataset distribution.

The result of pre-processing and feature engineering is a matrix of 126 records × 23 processed features that is used in the statistical analysis, feature selection and model development.

### Statistical analysis of the processed dataset

To determine whether the dataset has been drawn from a normally distributed population the normality of each feature was assessed using the Kolmogorov–Smirnov and Shapiro–Wilk tests. To identify the between-group differences between those having a poor vs. good surgical outcome, a Mann–Whitney U test was performed on the ordinal data and a Chi-square test for the categorical data. A correlation analysis was performed to identify co-linearities within the dataset using a Spearman’s rank correlation measuring the strength of association between pairs of features within the dataset. An alpha threshold on 0.05 was set for all statistical tests within the analysis and all tests were two-sided.

### Identifying candidate feature sets for the prediction of surgical outcomes

To identify a small set of clinically relevant features that are predictive of the surgical outcomes of CECS, feature selection was applied. Aside from identifying features relevant to the classification problem, feature selection reduces the dimensionality of the dataset, simplifying the classification problem, thereby improving model stability and generalisability. Feature selection was performed using a filter-based genetic algorithm and the knowledge of clinical experts.

A Tabu Asexual Genetic Algorithm (TAGA)^28^ was used to generate 9 feature sets (Supplementary Table S3). TAGA takes an *m* × *n* matrix, where *m* is the number of samples and *n* is the number of features and calculates a Fisher’s score. The top 12 features, based on their Fisher score, were entered into a genetic-based combinatorial optimisation process, outputting the desired number of features ($\lambda$) that maximise the relevant information within the selected features towards the classification target, whilst reducing redundancy. This process was conducted for $\lambda = 2, 3, \dots , 10$. The benefit of applying TAGA as opposed to other genetic-based feature selectors is that TAGA applies a tabu list, avoiding the algorithm getting stuck in a locally optimal solution, by storing a fixed amount of recently tested mutation swaps to be avoided in future mutations. Furthermore, the absence of a classifier in the selection process results in a set of features that are not biased towards any specific classifier, thus improving generalisability. Given that genetic algorithms can produce different results each time they are run, this process was repeated 30 times, outputting the selected $\lambda$ features for each iteration. Thereafter, the selection of final subsets of features for each cardinality was determined by calculating the relative frequency of each feature occurring in the selected subset and selecting those with the highest relative frequency.

A final set of potential features were created in consultation with the clinical team at DMRC. The team was presented with the list of features appearing in the optimally performing TAGA and statistically-derived feature sets and asked to create a combination of features that they would deem ‘sensible’ for the prediction of surgical outcomes (STAT + TAGA + Expert).

### Model development and evaluation

To identify which machine learning model would be most suitable for the task of predicting the surgical outcome, 8 classifiers were investigated. These models were logistic regression, support vector machines (SVM) and K-nearest neighbours (KNN), ensembled approaches (random forest, extreme gradient boosting (XGBoost), ensembled-KNN and ensembled-SVM), and a sequential model.

To evaluate the performance of each classifier, a repeated nested cross-validation was applied, as described in Algorithm 1. Firstly, inputs are defined, including the dataset, *A*, comprised of an $m \times n$ matrix, where *m* is the number of records in the training set and *n* is the number of features (Line 1) and a set of *H* hyperparameter combinations are specified for each model (Line 2). Dataset *A* is then split into 5 stratified folds to ensure that each fold has a similar distribution of groups as the whole dataset (Line 5). The number of folds was chosen due to the minority class comprising only 23% out of 126 records. Therefore, to have sufficient records of good surgical outcomes included in the test and validation sets to adequately evaluate a model in terms of sensitivity, fivefolds were chosen. Furthermore, fivefolds was viewed as a means of managing the bias associated with fewer folds and the variance associated with a large number of folds.

For each fold in the fivefold cross-validation, onefold is selected as the test set (Line 7), meaning its data will not be used to train or tune the model. Thereafter, hyperparameter tuning commences in the form of a fourfold stratified cross-validation, training a model on three of the remaining folds and validating the performance of hyperparameters *h* on the fourth remaining fold. Hyperparameter tuning was performed using a grid search and the optimal hyperparameter settings of each model are shown in Supplementary Table S5. Following the fourfold cross-validation, the mean performance overall fourfolds is calculated for each hyperparameter combination (Line 13), and the optimal combination is identified (Line 15). Performance was measured in terms of mean AUC on the validation set. Subsequently, the model is then retrained on all four folds using the optimal hyperparameters (Line 18), and tested on the fifth fold that was held out at the beginning of the outer loop (Line 19). The outer loop continues until each of the fivefolds has acted as the test set. This process is repeated 30 times to capture the variation in performance of an optimal model, resulting in 150 different train and test sets being evaluated for each classifier (Line 22). Random seeds were employed to ensure that the train and test sets were identical for all models, to enable a fair comparison between models (Line 3). 
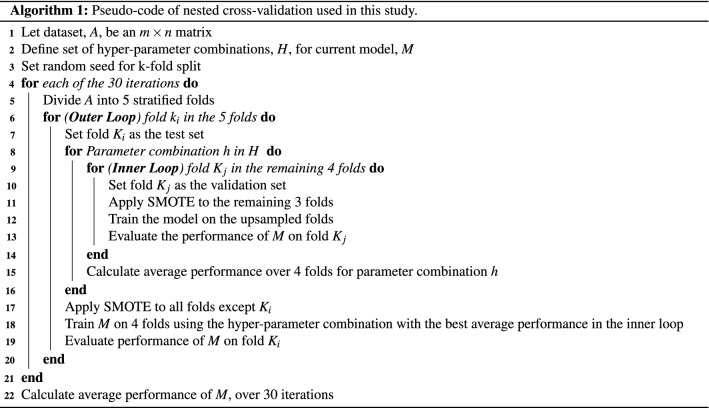


To tackle class imbalance, having only 29 records of patients with a good surgical outcome vs 97 records of patients with a poor surgical outcome, the minority class of the training set, i.e. records of patients with good surgical outcomes, was up-sampled using a synthetic minority over-sampling technique (SMOTE)^63^, as shown in lines 9 and 15 of Algorithm 1. SMOTE was not applied to the whole dataset to limit data leaking from the training set to the testing set. Furthermore, SMOTE was not applied to the testing set because the test set would no longer reflect the true distribution that exists in real life. The classification performance of each model, with and without the use of SMOTE is presented in Tables 1 and 2, respectively. It is clear that without SMOTE a highly imbalanced classifier results, characterised by high specificity and low sensitivity, meaning the classifiers are unable to accurately classify the minority class compare to the majority class in the dataset.

The performance of each classifier and feature set was measured using a number of evaluation metrics. The metrics employed included: accuracy, sensitivity, specificity and area under the receiver operating characteristic (ROC) curve (AUC), optimal false positive rate (FPR) and optimal true positive rate (TPR).

Accuracy is defined as the ratio of correct predictions compared to incorrect predictions. Sensitivity is defined as the percentage of positive cases being correctly identified. Specificity is defined as the percentage of negative cases being correctly identified. Accuracy, sensitivity and specificity are calculated by:

1 $$\begin{aligned} Accuracy= & {} \frac{TP+TN}{{TP + FP + TN + FN} } \end{aligned}$$

2 $$\begin{aligned} Sensitivity= & {} \frac{TP}{{TP + FN} } \end{aligned}$$

3 $$\begin{aligned} Specificity= & {} \frac{TN}{TN + FP} \end{aligned}$$

where *TP* refers to a patient who returned to work being predicted as a returning to work, *FN* refers to a patient who returned to work being predicted as not returning to work, *FP* refers to a patient who did not return to work being predicted as a returning to work and *TN* refers to a patient who did not return to work being predicted as a returning to work.

A Receiver Operating Characteristic (ROC) curve was constructed and the optimal operating point was identified as the intersection of the true positive rate and 1 minus the false positive rate. The false and true positive rates at the optimal operating point were identified as the model’s FPR and TPR, respectively. Lastly, the area under the ROC curve (AUC) was recorded as it reflects the model’s capability of discriminating between patients returning to work and those who do not.

A statistical comparison between models was conducted for each performance metric using Friedman’s paired samples tests to identify whether performance in each metric significantly differed between models. If a significant difference was found, a post-hoc analysis was conducted using a Wilcoxon signed-rank test, adjusting the significance threshold using the Bonferroni correction, to establish between which pair of models differences were significant. The analysis was conducted on the outputs of the outer loop of the nested cross-validation, reflecting model performance on unseen data. Given that a fivefold outer loop, repeated 30 times, was used, the classification performance of each model on 150 test sets was compared across the 6 performance metrics.

### Model interrogation methods

The intention of the model interrogation stage was employed to go beyond mean measures of overall model performance, digging deeper into identifying which model was best suited for the current classification problem. Model interrogation involved characterising each record from a difficulty perspective, then identifying sources of difficulty using associative testing and establishing which models are best suited to mitigate the effects of said sources of difficulty. To characterise a record in terms of its difficulty, an instance hardness score was calculated, reflecting the number of trialled classifiers capable of correctly predicting the outcome of each record. Instance hardness was derived using (4), as proposed by Smith et al.^25^:

4 $$\begin{aligned} instance\,hardness(x) = \frac{\sum _{i}^{N}incorrect(CL_i,x)}{N} \end{aligned}$$

where *x* refers to a record in the dataset, *N* refers to the number of classifiers and the function $incorrect(CL_i,x)$ returns 1 if the record *x* is incorrectly classified by classifier $CL_i$.

To determine why a record may have been misclassified, from a data perspective, four instance-level hardness measures were calculated: K-disagreeing neighbours (KDN), disjunct size (DS), disjunct class percentage (DCP) and class-likelihood percentage (CLP). The hardness measures are calculated as follows:

KDN measures the local overlap of the nearest neighbours of each instance, KDN represents the percentage of neighbours that do not share the same class as the current instance, and is derived using (5).

5 $$\begin{aligned} KDN(x) = \frac{|\{y : y \in KNN(x) \wedge t(y) \ne t(x)\}|}{k} \end{aligned}$$

DS measures the complexity of the decision boundary, and is derived using (6).

6 $$\begin{aligned} DS(x) = \frac{|disjunct(x)|-1}{max_{y\in D}|disjunct(y)|-1} \end{aligned}$$

DCP represents the overlap of an instance based on a subset of its features, and is derived using (7).

7 $$\begin{aligned} DCP(x) = \frac{|\{z:z \in disjunct(x) \wedge t(z) = t(x)\}|}{|disjunct(x)|} \end{aligned}$$

CLD represents the global overlap of an instance against the rest of the dataset using all features, and is derived using (8).

8 $$\begin{aligned} CLD(x) = CL(x, t(x)) - \underset{y \in Y - t(x)}{argmaxCL(x,y)} \end{aligned}$$

where the function *KNN*(*x*) returns the nearest *k* neighbours to record *x*, *t*(*x*) returns the class label of record *x*, the function *disjunct*(*x*) returns the disjunct that covers record *x*, *D* refers to the entire dataset, *Y* represents the number of classes in the dataset, *y* represents all records returned by the functions *KNN*(*x*) and *disjunct*(*x*) and *z* represents the records returned by the functions KNN(x) and disjunct(x) belonging to the same class as record *x*, the function *CL*(*x*) returns the probability of an instance belonging to a certain class, and is derived using Eq. (9):

9 $$\begin{aligned} CL(x) = CL(x,t(x)) = \prod _{i}^{|x|}P(x_i|t(x)) \end{aligned}$$

where |*x*| is the number of features of instance *x* and $x_i$ is the value of *x*’s *i*th feature.

To establish which harness measures best explain the difficulty of each record within the dataset, Spearman’s correlations were used, identifying the strength of association between instance hardness and each hardness measure. The measures with the largest correlation coefficients were carried forward to establish which model was best suited at handling the negative effects of each measure on classification performance.

To examine each model’s performance on records of high and low difficulty, AUC was calculated using the mean probability predicted for each record from the 30 iterations of the nested cross-validation, beginning with a set of records with low hardness measure scores. Incrementally, records with greater hardness measure scores were added until the entire dataset was included, at which point the records with the lowest hardness measure scores were removed until AUC was unable to be calculated due to the absence of one group from the dataset. In applying this approach, how each model performs on records with the characteristics associated with increased difficulty can be established. Therefore enabling an informed decision as to which classifier is most appropriate for deployment.

### Performance comparison with automated machine learning frameworks

The classification performance of the proposed framework was compared to three automated machine learning frameworks; Auto-Sklean^33^, TPOT^32^ and AutoPrognosis^34^. Both Auto-Sklearn and TPOT use frequentist tree-based structures to model the sparsity of a pipeline’s hyperparameter space. To optimise the derived pipeline, Auto-Sklearn applies Bayesian optimisation using tree-based heuristics, TPOT uses a tree-based genetic approach, and AutoPrognosis uses Gaussian process-based Bayesian optimisation with structured kernel learning. Both Auto-Sklearn and AutoPrognosis automate the processes of missing data imputation, data pre-processing, feature selection, and classifier selection and tuning (Supplementary Table S8). Therefore, for both frameworks, the raw, unprocessed data was input into the frameworks. TPOT performs all aforementioned processes with the exception of missing data imputation, therefore, the raw data was imputed before it was input into the TPOT framework. Imputation was performed using group-level median imputation, as performed in AITIA. All frameworks were evaluated using the same approach that was applied for evaluating the performance of AITIA as described in the “Model development and evaluation” section of the ‘Online methods’. To compare the framework proposed in this study with the automated frameworks, a pairwise analysis was carried out on the performance of each framework on the 150 test sets, across all 6 evaluation metrics, using Wilcoxon signed-ranks tests.

## Supplementary Information

Supplementary Tables.

## Data Availability

*Accession codes* The code for performing the model interrogation proposed in this study is publicly available at https://github.com/andrewhouston113/Machine-Learning-Model-Interrogation.
