# Author Correction: Predicting surgical outcomes for chronic exertional compartment syndrome using a machine learning framework with embedded trust by interrogation strategies

**DOI:** 10.1038/s41598-022-06176-w

**Published:** 2022-02-01

**Authors:** Andrew Houston, Georgina Cosma, Phillipa Turner, Alexander Bennett

**Affiliations:** 1 School of Computer Science, Loughborough University, Loughborough, LE11 3TU UK; 2 Academic Department of Military Rehabilitation, Defence Medical Services, Loughborough, LE12 5QW UK; 3 Centre for Lower-Limbs Rehabilitation, Defence Medical Services, Loughborough, LE12 5QW UK; 4 Imperial College London, National Heart and Lung Institute, London, SW7 2BU UK; 5 School of Sport, Exercise and Health Sciences, Loughborough University, Loughborough, LE11 3TU UK

Correction to: *Scientific Reports* 10.1038/s41598-021-03825-4, published online 20 December 2021

The original version of this Article contained an error in the spelling of the authors Andrew Houston, Georgina Cosma, Phillipa Turner and Alexander Bennett which were incorrectly given as Houston Andrew, Cosma Georgina, Turner Phillipa and Bennett Alexander. The original Article has been corrected.

