# Supplementary material for: Predicting surgical outcomes for chronic exertional compartment syndrome using a machine learning framework with embedded trust by interrogation strategies
Source: Sci Rep. 2021 Dec 20;11:24281. doi: 10.1038/s41598-021-03825-4 (PMC8688508; doi:10.1038/s41598-021-03825-4)
Supplement: Supplementary file 1 — Supplementary Tables. [file 41598_2021_3825_MOESM1_ESM.pdf]

Supplemental Files

		Kolmogorov–Smirnov test		Shapiro-Wilk	
**Feature**	**Outcome**	**Statistic**	**Sig.**	**Statistic**	**Sig.**
**Age**	0	0.15	0.00	0.97	0.03
	1	0.15	0.20	0.95	0.32
**Job Rank**	0	0.24	0.00	0.69	0.00
	1	0.32	0.00	0.63	0.00
**Height**	0	0.07	0.20	0.99	0.87
	1	0.18	0.09	0.95	0.37
**Weight**	0	0.07	0.20	0.98	0.14
	1	0.13	0.20	0.98	0.89
**BMI**	0	0.06	0.20	0.99	0.58
	1	0.15	0.20	0.94	0.26
$\mathbf{BP}_{systolic}$	0	0.08	0.20	0.98	0.40
	1	0.14	0.20	0.91	0.06
$\mathbf{BP}_{diastolic}$	0	0.08	0.20	0.98	0.23
	1	0.15	0.20	0.97	0.64
**MAP**	0	0.10	0.06	0.98	0.18
	1	0.12	0.20	0.98	0.86
**Smoker**	0	0.41	0.00	0.62	0.00
	1	0.45	0.00	0.44	0.00
**Alcohol**	0	0.25	0.00	0.68	0.00
	1	0.18	0.07	0.88	0.02
**FAAM**	0	0.10	0.03	0.96	0.02
	1	0.11	0.20	0.97	0.77
**EILP**	0	0.11	0.02	0.97	0.05
	1	0.17	0.14	0.95	0.39
**Chronicity**	0	0.10	0.06	0.90	0.00
	1	0.24	0.00	0.83	0.00
**TTP**	0	0.19	0.00	0.80	0.00
	1	0.28	0.00	0.67	0.00
**TTD**	0	0.12	0.01	0.93	0.00
	1	0.25	0.00	0.81	0.00
**TTT**	0	0.10	0.04	0.94	0.00
	1	0.28	0.00	0.80	0.00
**Wait Time**	0	0.35	0.00	0.59	0.00
	1	0.23	0.01	0.87	0.01
**TDG**	0	0.19	0.00	0.81	0.00
	1	0.31	0.00	0.61	0.00
**Co-morbidities**	0	0.49	0.00	0.49	0.00
	1	0.54	0.00	0.24	0.00
**Prior Injuries**	0	0.44	0.00	0.51	0.00
	1	0.49	0.00	0.50	0.00
**Prior Surgeries**	0	0.49	0.00	0.48	0.00
**Prior IR**	0	0.27	0.00	0.76	0.00
	1	0.49	0.00	0.50	0.00

Table S.1: Results of the normality tests applied to the dataset. An outcome of 0 refers to patients who did not return to full medical deployability, 1 refers to those who did. IR = Inpatient Rehabilitation.

ID	Feature	Groups	Did not RTW	RTW	Statistic	Sig.
1	Age	≤ 25	13	10	-1.972[a]	0.049*
		$25 < \text{age} \leq 30$	52	11		
		>30	32	8		
2	Job Rank	0 (eg. private)	37	8	-0.878[a]	0.380
		1 (eg. lance corporal)	32	10		
		2+ (eg. corporal major)	28	11		
3	Height	< 170	35	3	-2.717[a]	0.007*
		$170 \leq \text{height} < 180$	40	13		
		≥ 180	22	13		
4	Weight	< 75	23	7	-0.774[a]	0.439
		$75 \leq \text{weight} < 85$	18	8		
		$85 \leq \text{weight} < 95$	27	6		
		≥ 95	29	8		
5	BMI	Normal weight ($18.5 \leq \text{BMI} < 25$)	18	12	-2.179[a]	0.029*
		Overweight ($25 \leq \text{BMI} < 30$)	48	11		
		Obese (≥ 30)	31	6		
6	$BP_{systolic}$	< 120	13	4	-0.127[a]	0.899
		$120 \leq BP_{systolic} < 130$	39	9		
		$130 \leq BP_{systolic} < 140$	29	13		
		≥ 140	16	3		
7	$BP_{diastolic}$	< 80	64	22	-1.033[a]	0.301
		$80 \leq BP_{diastolic} < 90$	26	7		
		≥ 90	7	0		
8	MAP	$60 \leq \text{MAP} < 100$	73	27	-1.992[a]	0.046
		≥ 100	24	2		
9	BP	Normal	11	3	-0.591[a]	0.554
		Elevated	31	7		
		Stage 1 high BP	46	16		
		Stage 2 high BP	9	3		
10	Smoking Status	Non-smoker	69	25	-1.585[a]	0.113
		Light smoker (1-9 cigs/day)	6	2		
		Moderate smoker (10-19 cigs/day)	17	1		
		Heavy smoker (20+ cigs/day)	5	1		
11	Alcohol Status	Non-binge drinker ($\leq$ 14 units/week)	80	24	-0.062[a]	0.950
		Binge drinker (>14 units/week)	17	5		
12	FAAM	Extremely Affected (< 20%)	33	8	-0.185[a]	0.853
		Very affected (20% to < 40%)	52	19		
		Moderately affected (40% to < 60%)	11	2		
		Slightly affected (60% to < 80%)	1	0		
13	EILP	Extremely affected (< 20%)	8	5	-2.868[a]	0.004*
		Very affected (20% to < 40%)	27	15		
		Moderately affected (40% to < 60%)	37	4		
		Slightly affected (60% to < 80%)	22	4		
		Unaffected (80% to < 100%)	3	1		

14	Chronicity	≤ 2 years	8	12	-4.071^a	< 0.001**
		2 years < chronicity ≤ 3 years	9	5		
		3 years < chronicity ≤ 4 years	17	4		
		4 years < chronicity ≤ 5 years	16	2		
		5 years < chronicity ≤ 6 years	10	4		
		6 years < chronicity ≤ 7 years	13	1		
		>7 years	24	1		
15	TTP	≤ 3 months	26	13	-1.664^a	0.096
		3 months < TTP ≤ 6 months	10	2		
		6 months < TTP ≤ 1 year	11	6		
		1 year < TTP ≤ 2 years	21	2		
		2 years < TTP ≤ 4 years	18	4		
		>4 years	11	2		
16	TTD	≤ 1 year	12	14	-4.579^a	< 0.001**
		1 year < TTT ≤ 2 years	15	9		
		2 years < TTT ≤ 3 years	12	2		
		3 years < TTT ≤ 4 years	22	0		
		4 years < TTT ≤ 5 years	13	3		
		>5 years	23	1		
17	TTT	≤ 1 year	7	7	-4.657^a	< 0.001**
		1 year < TTD ≤ 2 years	13	14		
		2 years < TTD ≤ 3 years	12	3		
		3 years < TTD ≤ 4 years	20	1		
		4 years < TTD ≤ 5 years	13	3		
		>5 years	31	1		
18	TDG	≤ 6 months	7	6	-3.628^a	< 0.001**
		6 months < TDG ≤ 1 year	20	9		
		1 year < TDG ≤ 2 years	24	11		
		2 years < TDG ≤ 3 years	15	2		
		>3 years	31	1		
19	Wait time	≤ 3 months	24	9	-1.200^a	0.230
		3 months < TDG ≤ 6 months	56	19		
		6 months < TDG ≤ 1 year	9	2		
		>1 year	8	0		
20	Prior injuries	Yes	78	27	0.113^b	0.807
		No	19	2		
21	Co-morbidities	Yes	73	23	2.351^b	0.102
		No	24	6		
22	Prior fasciotomies	Yes	82	29	4.865^b	0.041*
		No	15	0		
23	Prior inpatient rehabilitation	Yes	43	21	6.133^b	0.018*
		No	54	8		

Table S.2: Data description of each input feature contained within the dataset and the results of the between group analysis. ^{a} Z score output by the Mann Whitney U test; ^{b} Chi-Squared test statistic

**Subset Name**	**Features**									
Stats 1	1	3	5	13	14	18	22	23		
Stats 2	1	3	5	13	16	18	22	23		
Stats 3	1	3	5	13	17	18	22	23		
Stats 4	3	12	14	18						
Stats 5	3	12	16	18						
Stats 6	3	12	17	18						
Stats 7	14	18								
Stats 8	16	18								
Stats 9	17	18								
TAGA 1	3	14								
TAGA 2	3	5	14							
TAGA 3	3	5	8	14						
TAGA 4	3	5	8	13	14					
TAGA 5	3	5	8	13	14	23				
TAGA 6	3	5	8	13	14	15	23			
TAGA 7	3	5	8	13	14	15	18	23		
TAGA 8	3	5	8	13	14	15	18	22	23	
TAGA 9	1	3	5	8	13	14	15	18	22	23
STAT+TAGA+Expert	3	8	12	16	18	22				

Table S.3: Feature sets derived using the results of the statistical analysis, the TAGA and Expert knowledge.

Subset	LR AUC	LR Rank	SVM AUC	SVM Rank	KNN AUC	KNN Rank	RF AUC	RF Rank	XGB AUC	XGB Rank	ESVM AUC	ESVM Rank	EKNN AUC	EKNN Rank	SEQ AUC	SEQ Rank
Stats 1	0.81	6	0.82	8	0.79	4	0.78	8	0.81	7	0.82	8	0.81	4	0.81	5
Stats 2	0.82	5	0.81	11	0.79	4	0.80	4	0.82	5	0.82	8	0.81	4	0.81	5
Stats 3	0.81	6	0.82	8	0.78	9	0.81	2	0.82	5	0.82	8	0.82	2	0.81	5
Stats 4	0.83	4	0.83	5	0.78	9	0.77	12	0.83	4	0.84	2	0.81	4	0.83	1
Stats 5	0.84	1	0.83	5	0.80	1	0.80	4	0.84	1	0.84	2	0.81	4	0.83	1
Stats 6	0.84	1	0.84	2	0.79	4	0.81	2	0.84	1	0.84	2	0.81	4	0.83	1
Stats 7	0.80	8	0.81	11	0.78	9	0.78	8	0.79	15	0.80	12	0.77	15	0.79	11
Stats 8	0.79	11	0.79	17	0.74	15	0.79	6	0.80	8	0.79	16	0.78	12	0.77	18
Stats 9	0.79	11	0.80	15	0.74	15	0.79	6	0.80	8	0.80	12	0.78	12	0.77	18
TAGA 1	0.79	11	0.78	18	0.74	15	0.75	17	0.79	15	0.77	19	0.76	18	0.78	16
TAGA 2	0.79	11	0.78	18	0.73	19	0.75	17	0.80	8	0.78	18	0.75	19	0.78	16
TAGA 3	0.79	11	0.80	15	0.74	15	0.74	19	0.80	8	0.79	16	0.77	15	0.79	11
TAGA 4	0.79	11	0.81	11	0.75	14	0.76	15	0.80	8	0.80	12	0.77	15	0.79	11
TAGA 5	0.79	11	0.82	8	0.76	13	0.77	12	0.79	15	0.81	11	0.79	11	0.79	11
TAGA 6	0.78	19	0.81	11	0.77	12	0.76	15	0.78	19	0.80	12	0.78	12	0.79	11
TAGA 7	0.80	8	0.84	2	0.79	4	0.78	8	0.80	8	0.83	6	0.81	4	0.81	5
TAGA 8	0.80	8	0.84	2	0.80	1	0.77	12	0.80	8	0.84	2	0.81	4	0.81	5
TAGA 9	0.79	11	0.83	5	0.79	4	0.78	8	0.79	15	0.83	6	0.82	2	0.81	5
STAT+TAGA+Expert	0.84	1	0.85	1	0.80	1	0.82	1	0.84	1	0.85	1	0.83	1	0.82	4

Table S.4: Results of the feature selection process identifying the optimal feature set for each classifier based on AUC performance on the test set. Cells highlighted in green reflect the feature set identified as being superior with respect to the model associated with that cell.

Classifiers	Hyperparameters
Logistic Regression	Solver: Liblinear<br>C: 0.25
SVM	Kernel: Linear<br>C: 0.45
KNN	Neighbours: 11<br>Weights: Uniform<br>Metric: Manhattan
Random Forest	Estimators: 20<br>Max Depth: 1
XGBoost	Learning Rate: 0.1<br>Max Depth: 1<br>Estimators: 20
Ensembled SVM	Max Features: 0.75<br>Max Samples: 0.7<br>Estimators: 10
Ensembled KNN	Max Features: 0.75<br>Max Samples: 0.7<br>Estimators: 10
Sequential	Dense Layers: 1 (units = 64)<br>Activation Function: Sigmoid

Table S.5: Optimal hyperparameter settings for each classifier as determined by the internal loop of the nested cross validation. Solver = optimisation algorithm; C = inverse of the regularisation strength; Kernel = the kernel type to be used, e.g. linear, polynomial; Neighbours = the number of nearest records to consider when making is prediction; Weights = how to weight neighbors when making a prediction; Metric = Distance calculation algorithm; Estimators = number of trees; Depth = maximum depth of the tree; Max Features = Number of features to draw to train each classifier; Dense layer units = dimensionality of the output space of the dense layer.

Model	Set	Acc	Sens	Spec	Auc	Fpr	Tpr
Logistic Regression	Train	0.80 ± 0.03	0.3 ± 0.11	0.95 ± 0.02	0.87 ± 0.03	0.17 ± 0.04	0.83 ± 0.04
	Validation	0.80 ± 0.06	0.31 ± 0.16	0.94 ± 0.05	0.84 ± 0.08	0.20 ± 0.10	0.81 ± 0.11
	Test	0.79 ± 0.05	0.28 ± 0.18	0.94 ± 0.05	0.84 ± 0.09	0.18 ± 0.10	0.81 ± 0.11
SVM	Train	0.84 ± 0.04	0.61 ± 0.21	0.91 ± 0.03	0.87 ± 0.03	0.16 ± 0.06	0.84 ± 0.06
	Validation	0.82 ± 0.06	0.54 ± 0.23	0.90 ± 0.07	0.84 ± 0.08	0.20 ± 0.10	0.80 ± 0.12
	Test	0.82 ± 0.06	0.62 ± 0.20	0.88 ± 0.07	0.85 ± 0.08	0.19 ± 0.11	0.82 ± 0.11
KNN	Train	0.83 ± 0.03	0.6 ± 0.16	0.90 ± 0.03	0.87 ± 0.03	0.19 ± 0.04	0.82 ± 0.05
	Validation	0.80 ± 0.06	0.52 ± 0.22	0.88 ± 0.07	0.83 ± 0.08	0.22 ± 0.08	0.80 ± 0.11
	Test	0.79 ± 0.07	0.49 ± 0.22	0.87 ± 0.07	0.83 ± 0.08	0.21 ± 0.08	0.81 ± 0.11
Random Forest	Train	0.77 ± 0.01	0.01 ± 0.03	1.00 ± 0.00	0.87 ± 0.03	0.20 ± 0.04	0.80 ± 0.04
	Validation	0.77 ± 0.02	0.00 ± 0.03	1.00 ± 0.01	0.82 ± 0.08	0.25 ± 0.09	0.75 ± 0.11
	Test	0.77 ± 0.02	0.00 ± 0.00	1.00 ± 0.00	0.82 ± 0.09	0.24 ± 0.10	0.75 ± 0.11
XGBoost	Train	0.77 ± 0.01	0.00 ± 0.00	1.00 ± 0.00	0.84 ± 0.03	0.21 ± 0.04	0.79 ± 0.04
	Validation	0.77 ± 0.01	0.00 ± 0.00	1.00 ± 0.00	0.82 ± 0.08	0.23 ± 0.10	0.78 ± 0.11
	Test	0.77 ± 0.02	0.00 ± 0.00	1.00 ± 0.00	0.83 ± 0.09	0.21 ± 0.11	0.78 ± 0.11
Ensembled SVM	Train	0.78 ± 0.02	0.03 ± 0.09	1.00 ± 0.01	0.86 ± 0.04	0.18 ± 0.06	0.81 ± 0.06
	Validation	0.77 ± 0.02	0.02 ± 0.08	0.99 ± 0.02	0.83 ± 0.08	0.22 ± 0.11	0.78 ± 0.12
	Test	0.77 ± 0.02	0.01 ± 0.05	0.99 ± 0.02	0.83 ± 0.09	0.20 ± 0.11	0.81 ± 0.11
Ensembled KNN	Train	0.81 ± 0.03	0.32 ± 0.23	0.96 ± 0.04	0.88 ± 0.03	0.18 ± 0.04	0.82 ± 0.04
	Validation	0.79 ± 0.05	0.24 ± 0.22	0.95 ± 0.06	0.83 ± 0.08	0.22 ± 0.10	0.79 ± 0.12
	Test	0.77 ± 0.05	0.27 ± 0.22	0.93 ± 0.07	0.83 ± 0.09	0.20 ± 0.09	0.80 ± 0.12
Sequential	Train	0.81 ± 0.03	0.34 ± 0.13	0.95 ± 0.02	0.87 ± 0.03	0.18 ± 0.04	0.82 ± 0.04
	Validation	0.79 ± 0.06	0.3 ± 0.18	0.94 ± 0.06	0.83 ± 0.08	0.21 ± 0.10	0.79 ± 0.11
	Test	0.79 ± 0.06	0.32 ± 0.18	0.92 ± 0.06	0.83 ± 0.08	0.20 ± 0.09	0.80 ± 0.10

Table S.6: Results of the nested cross validation for each model, trained using their respective optimal feature set, without the implementation of SMOTE. Cells highlighted in green reflect the framework identified as being superior with respect to the outcome measure associated with that cell.

Table S.7: Pairwise comparisons between each model for all performance metrics. Bonferroni's correction was applied to the p-value resulting in an adjusted alpha of < 0.0018.

**Accuracy**	**Logistic Regression**	**SVM**	**KNN**	**Random Forest**	**XGBoost**	**Ensembled SVM**	**Ensembled KNN**
**SVM**	Z = -.448<br>p = 0.654						
**KNN**	Z = -8.393<br>p < 0.001*	Z = -8.230<br>p < 0.001*					
**Random Forest**	Z = -4.672<br>p < 0.001*	Z = -4.248<br>p < 0.001*	Z = -5.560<br>p < 0.001*				
**XGBoost**	Z = -1.784<br>p = 0.074	Z = -1.119<br>p = 0.263	Z = -7.906<br>p < 0.001*	Z = -4.096<br>p < 0.001*			
**Ensembled SVM**	Z = -.028<br>p = 0.978	Z = -.052<br>p = 0.959	Z = -8.702<br>p < 0.001*	Z = -4.780<br>p < 0.001*	Z = -1.329<br>p = 0.184		
**Ensembled KNN**	Z = -6.225<br>p < 0.001*	Z = -6.224<br>p < 0.001*	Z = -4.460<br>p < 0.001*	Z = -2.231<br>p = 0.026	Z = -5.507<br>p < 0.001*	Z = -6.770<br>p < 0.001*	
**Sequential**	Z = -5.625<br>p < 0.001*	Z = -3.214<br>p = 0.001*	Z = -6.774<br>p < 0.001*	Z = -1.480<br>p = 0.139	Z = -2.676<br>p = 0.007	Z = -3.302<br>p = 0.001*	Z = -3.703<br>p < 0.001*

**Sensitivity**	**Logistic Regression**	**SVM**	**KNN**	**Random Forest**	**XGBoost**	**Ensembled SVM**	**Ensembled KNN**
**SVM**	Z = -.546<br>p = 0.585						
**KNN**	Z = -.070<br>p = 0.944	Z = -.829<br>p = 0.407					
**Random Forest**	Z = -4.591<br>p < 0.001*	Z = -4.142<br>p < 0.001*	Z = -4.959<br>p < 0.001*				
**XGBoost**	Z = -6.449<br>p < 0.001*	Z = -5.642<br>p < 0.001*	Z = -5.990<br>p < 0.001*	Z = -1.647<br>p = 0.1			
**Ensembled SVM**	Z = -.528<br>p = 0.598	Z = -2.431<br>p = 0.015	Z = -.570<br>p = 0.569	Z = -5.034<br>p < 0.001*	Z = -6.517<br>p < 0.001*		
**Ensembled KNN**	Z = -2.170<br>p = 0.03	Z = -3.254<br>p = 0.001*	Z = -2.419<br>p = 0.016	Z = -6.388<br>p < 0.001*	Z = -7.773<br>p < 0.001*	Z = -1.791<br>p = 0.073	
**Sequential**	Z = -3.664<br>p < 0.001*	Z = -1.829<br>p = 0.067	Z = -3.159<br>p = 0.002	Z = -2.594<br>p = 0.009	Z = -4.081<br>p < 0.001*	Z = -2.830<br>p = 0.005	Z = -4.428<br>p < 0.001*

**Specificity**	**Logistic Regression**	**SVM**	**KNN**	**Random Forest**	**XGBoost**	**Ensembled SVM**	**Ensembled KNN**
**SVM**	Z = -.683<br>p = 0.495						
**KNN**	Z = -8.956<br>p < 0.001*	Z = -8.509<br>p < 0.001*					
**Random Forest**	Z = -2.113<br>p = 0.035	Z = -1.605<br>p = 0.108	Z = -7.216<br>p < 0.001*				
**XGBoost**	Z = -3.890<br>p < 0.001*	Z = -3.302<br>p = 0.001*	Z = -9.604<br>p < 0.001*	Z = -5.002<br>p < 0.001*			
**Ensembled SVM**	Z = -1.061<br>p = 0.289	Z = -.540<br>p = 0.589	Z = -8.833<br>p < 0.001*	Z = -1.237<br>p = 0.216	Z = -4.417<br>p < 0.001*		
**Ensembled KNN**	Z = -7.442<br>p < 0.001*	Z = -6.878<br>p < 0.001*	Z = -4.099<br>p < 0.001*	Z = -5.421<br>p < 0.001*	Z = -8.601<br>p < 0.001*	Z = -7.196<br>p < 0.001*	
**Sequential**	Z = -4.668<br>p < 0.001*	Z = -2.544<br>p = 0.011	Z = -7.793<br>p < 0.001*	Z = -.443<br>p = 0.658	Z = -5.943<br>p < 0.001*	Z = -2.110<br>p = 0.035	Z = -6.153<br>p < 0.001*

AUC	Logistic Regression	SVM	KNN	Random Forest	XGBoost	Ensembled SVM	Ensembled KNN
SVM	Z = -4.025 $p < 0.001$*						
KNN	Z = -6.919 $p < 0.001$*	Z = -8.340 $p < 0.001$*					
Random Forest	Z = -2.860 p = 0.004	Z = -4.990 $p < 0.001$*	Z = -4.464 $p < 0.001$*				
XGBoost	Z = -1.376 p = 0.169	Z = -4.174 $p < 0.001$*	Z = -5.899 $p < 0.001$*	Z = -2.373 p = 0.018			
Ensembled SVM	Z = -3.448 p = 0.001*	Z = -.208 p = 0.835	Z = -7.668 $p < 0.001$*	Z = -4.887 $p < 0.001$*	Z = -4.559 $p < 0.001$*		
Ensembled KNN	Z = -3.341 p = 0.001*	Z = -6.493 $p < 0.001$*	Z = -5.953 $p < 0.001$*	Z = -.244 p = 0.807	Z = -2.270 p = 0.023	Z = -5.342 $p < 0.001$*	
Sequential	Z = -4.945 $p < 0.001$*	Z = -6.119 $p < 0.001$*	Z = -5.884 $p < 0.001$*	Z = -1.032 p = 0.302	Z = -1.641 p = 0.101	Z = -5.306 $p < 0.001$*	Z = -1.298 p = 0.194

FPR	Logistic Regression	SVM	KNN	Random Forest	XGBoost	Ensembled SVM	Ensembled KNN
SVM	Z = -.160 p = 0.873						
KNN	Z = -5.746 $p < 0.001$*	Z = -5.075 $p < 0.001$*					
Random Forest	Z = -5.702 $p < 0.001$*	Z = -5.102 $p < 0.001$*	Z = -.829 p = 0.407				
XGBoost	Z = -2.170 p = 0.03	Z = -1.433 p = 0.152	Z = -3.941 $p < 0.001$*	Z = -5.188 $p < 0.001$*			
Ensembled SVM	Z = -.475 p = 0.635	Z = -.184 p = 0.854	Z = -5.266 $p < 0.001$*	Z = -5.623 $p < 0.001$*	Z = -1.776 p = 0.076		
Ensembled KNN	Z = -2.246 p = 0.025	Z = -2.170 p = 0.03	Z = -3.948 $p < 0.001$*	Z = -4.206 $p < 0.001$*	Z = -.578 p = 0.563	Z = -2.318 p = 0.02	
Sequential	Z = -4.065 $p < 0.001$*	Z = -2.738 p = 0.006	Z = -3.189 p = 0.001*	Z = -3.529 $p < 0.001$*	Z = -1.020 p = 0.308	Z = -3.349 p = 0.001*	Z = -.510 p = 0.61

TPR	Logistic Regression	SVM	KNN	Random Forest	XGBoost	Ensembled SVM	Ensembled KNN
SVM	Z = -.603 p = 0.546						
KNN	Z = -3.659 $p < 0.001$*	Z = -4.223 $p < 0.001$*					
Random Forest	Z = -3.304 p = 0.001*	Z = -3.451 p = 0.001*	Z = -1.102 p = 0.27				
XGBoost	Z = -2.564 p = 0.01	Z = -2.483 p = 0.013	Z = -2.101 p = 0.036	Z = -1.182 p = 0.237			
Ensembled SVM	Z = -1.145 p = 0.252	Z = -1.814 p = 0.07	Z = -2.690 p = 0.007	Z = -2.002 p = 0.045	Z = -1.170 p = 0.242		
Ensembled KNN	Z = -1.394 p = 0.163	Z = -1.732 p = 0.083	Z = -3.056 p = 0.002	Z = -1.950 p = 0.051	Z = -1.028 p = 0.304	Z = -.236 p = 0.814	
Sequential	Z = -3.136 p = 0.002	Z = -2.608 p = 0.009	Z = -1.719 p = 0.086	Z = -.682 p = 0.495	Z = -.506 p = 0.613	Z = -1.822 p = 0.068	Z = -1.237 p = 0.216

Framework	Imputation	Pre-Processing	Feature Selection	Classifier Selection	Tuning
TPOT [32]	Not included	Employs standard and robust scaler operators polynomial feature generation and randomised PCA.	Uses recursive feature elimination, which either selects the top k features, selects the top n percent of features or removes features that do not meet a minimum variance threshold.	Implements 13 classifiers incorporating a range of individual and ensemble tree-based supervised models.	Genetic programming is applied to not only tune pipeline parameters, but also hyperparameters of the selected classifier.
Auto-Sklearn [33]	Incorporates both median and mean imputation.	14 feature pre-processing methods are incorporated, including decomposition using ICA and PCA, one-hot encoding and feature scaling.	Feature selection can be performed using random kitchen sinks, PCA or by selecting the n best features.	Implements 15 individual and ensemble tree-based supervised models. Furthermore, Auto-Sklearn automatically creates ensembles of models using Bayesian optimisation.	Optimisation and tuning of the generated pipeline and associated hyperparameters is performed using Bayesian optimisation.
Auto-Prognosis [34]	8 data imputation methods are included, encompassing mean and median imputation through to forest-based imputation.	Applies a number of pre-processing techniques including PCA, a polynomial feature generator for creating interaction features and feature agglomeration.	Feature selection is performed using random kitchen sinks, PCA or by selecting the n best features using selection rates.	Implements 20 individual and ensemble tree-based supervised models. Furthermore, Auto-Sklearn automatically creates ensembles of models using Bayesian model averaging.	Batched Bayesian optimisation with structured kernel learning is used to optimise and tuning of the generated pipeline and associated hyperparameters.

Table S.8: A general description of the processed included in each of the automated frameworks used in this study.
